# Initial Patient Characteristics Associated With Ineligibility for Second‐Line Therapy After Progression on First‐Line Osimertinib in *EGFR* ‐Mutated Non‐Small Cell Lung Cancer

**DOI:** 10.1111/1759-7714.70192

**Published:** 2025-11-17

**Authors:** Hiroaki Kodama, Haruyasu Murakami, Nobuaki Mamesaya, Haruki Kobayashi, Kazushige Wakuda, Ryo Ko, Akira Ono, Hirotsugu Kenmotsu, Tateaki Naito, Tetsuo Shimizu, Yasuhiro Gon, Toshiaki Takahashi

**Affiliations:** ^1^ Division of Thoracic Oncology Shizuoka Cancer Center Shizuoka Japan; ^2^ Department of Internal Medicine, Division of Respiratory Medicine Nihon University School of Medicine Tokyo Japan

**Keywords:** CNS metastasis, EGFR‐TKI, NSCLC, older patients, osimertinib

## Abstract

**Background:**

Osimertinib, an irreversible third‐generation epidermal growth factor receptor tyrosine kinase inhibitor (EGFR‐TKI), improves survival in patients with *EGFR*‐mutated non‐small‐cell lung cancer (NSCLC). However, a substantial proportion of patients do not proceed to subsequent therapy (ST). This study aimed to identify patient characteristics associated with ineligibility for post‐osimertinib therapy.

**Methods:**

We retrospectively enrolled patients with *EGFR*‐mutated NSCLC who received first‐line osimertinib monotherapy between January 2016 and June 2023. Patients were categorized into a ST group and a non‐subsequent therapy (NST) group. Baseline characteristics, treatment outcomes, and reasons for treatment discontinuation were documented.

**Results:**

Of 94 patients, 57 (60.6%) received ST, whereas 37 (39.4%) did not. The ST and NST groups significantly differed in age (*p* < 0.01) and prevalence of baseline central nervous system (CNS) metastases (24.1% vs. 54.1%, *p* < 0.01). The overall response rate to first‐line osimertinib was similar (63.2% vs. 56.8%, *p* = 0.71), as was median progression‐free survival (16.4 [95% confidence interval, CI: 12.4–21.4] vs. 16.1 months [95% CI: 9.8–19.3]; *p* = 0.24). The primary reason for not receiving subsequent treatment was deterioration in performance status, most often due to worsening pulmonary disease or CNS metastasis. In patients with baseline CNS metastasis, the main cause was also progression of CNS metastasis.

**Conclusions:**

Advanced age and baseline CNS metastasis are significant barriers to receiving second‐line therapy after osimertinib monotherapy. Alternate first‐line treatment strategies may be warranted for such patients.

## Graphical abstract

In patients receiving osimertinib monotherapy, advanced age over 75 years and baseline CNS metastases were found to be significant barriers to subsequent treatment. Initiating osimertinib monotherapy in these patients may limit future treatment opportunities; hence, the potential for transitioning to subsequent treatments should be considered when selecting initial therapy.

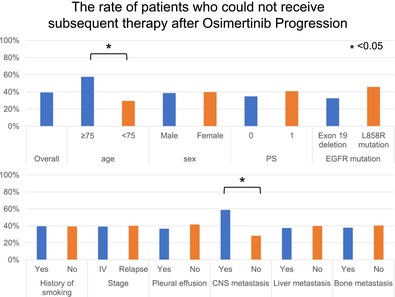

## 1 Introduction

The advent of epidermal growth factor receptor tyrosine kinase inhibitors (EGFR‐TKIs) has significantly improved survival outcomes in patients with advanced *EGFR*‐mutated non‐small cell lung cancer (NSCLC). Osimertinib, an irreversible third‐generation EGFR‐TKI, has been the standard first‐line treatment for *EGFR*‐mutated NSCLC since the publication of the FLAURA trial results [1, 2]. However, almost all patients eventually develop acquired resistance, with a median progression‐free survival (PFS) of 18.9 months [1]. To overcome this resistance, several strategies have been explored, including combining chemotherapy with osimertinib, integrating bispecific antibodies with EGFR‐TKIs, and optimizing post‐osimertinib therapies [3, 4, 5].

The FLAURA2 trial demonstrated that osimertinib combined with chemotherapy significantly improved the median PFS to 25.5 months, compared with 16.7 months for osimertinib monotherapy [3]. Similarly, combination therapy with amivantamab and lazertinib yielded a median PFS of 23.7 months, outperforming the 16.6 months observed with osimertinib monotherapy [5]. Despite these advances, improved outcomes are accompanied by higher toxicity and greater healthcare resource utilization. Moreover, such approaches may constitute overtreatment in patients who derive sufficient benefit from osimertinib monotherapy [6]. In contrast, some patients treated with osimertinib monotherapy are unable to transition to second‐line therapy following disease progression, indicating that monotherapy may not be optimal for this subgroup [1, 3].

Determining the most effective treatment strategy for advanced *EGFR*‐mutated NSCLC, therefore, remains a clinical challenge, underscoring the need to define an ideal treatment sequence. In this study, we aimed to identify the risk factors associated with failure to receive second‐line therapy following progression on first‐line osimertinib.

## 2 Materials and Methods

### 2.1 Patients

We retrospectively reviewed the records of 238 patients with advanced *EGFR*‐mutated NSCLC who received first‐line osimertinib at Shizuoka Cancer Center between January 2016 and June 2023. Eligible patients had metastatic or recurrent non‐squamous NSCLC, an Eastern Cooperative Oncology Group performance status (PS) score of 0–1, and harbored either an exon 19 deletion or L858R EGFR mutation. *EGFR* mutations were detected using one of the following assays: Cobas *EGFR* mutation test v2 (Roche Molecular Systems, Pleasanton, CA), Oncomine Comprehensive Assay v3 (Thermo Fisher Scientific, MA), or AmoyDx Pan Lung Cancer PCR Panel (Amoy Diagnostics, Xiamen, China).

Patients were categorized into two groups based on post‐progression treatment: those who received subsequent therapy (ST group) and those who did not (non‐subsequent therapy, NST group). The ST group included patients who received subsequent systemic anticancer treatment with cytotoxic chemotherapy, immune checkpoint inhibitors, or molecularly targeted therapies. Baseline characteristics and treatment responses before and after osimertinib administration were compared between the two groups. Additionally, reasons for treatment discontinuation in the NST group were examined to identify potential predictors of ineligibility for second‐line therapy. Beyond PD treatment with osimertinib after RECIST PD was defined as continuation of initial therapy. Palliative treatments such as palliative radiation therapy were not considered in the classification.

### 2.2 Statistical Analysis

PFS was defined as the time from initiation of osimertinib to disease progression or censoring at the last follow‐up. Overall survival (OS) was defined as the time from initiation of osimertinib to the date of death. Differences in patient characteristics between the ST and NST groups were assessed using Fisher's exact test for categorical variables and the Mann–Whitney *U* test for continuous variables. We used Cox's proportional hazards analysis for the multivariate analysis. Kaplan–Meier analysis was performed to evaluate OS and PFS, with statistical significance set at *p* < 0.05. All analyses were conducted using EZR software version 1.6‐3 (Saitama Medical Center, Jichi Medical University), which incorporates biostatistical functions into the R Commander interface.

### 2.3 Ethical Considerations

This study was approved by the Institutional Review Board of Shizuoka Cancer Center (J2024‐224‐2024‐1‐3) and conducted in accordance with the principles of the Declaration of Helsinki. Informed consent was obtained in the form of an opt‐out via the hospital website and onsite notices. Patients who declined participation were excluded.

## 3 Results

### 3.1 Characteristics of Patients at the Time of Initiation of Osimertinib Monotherapy

**FIGURE 1:**
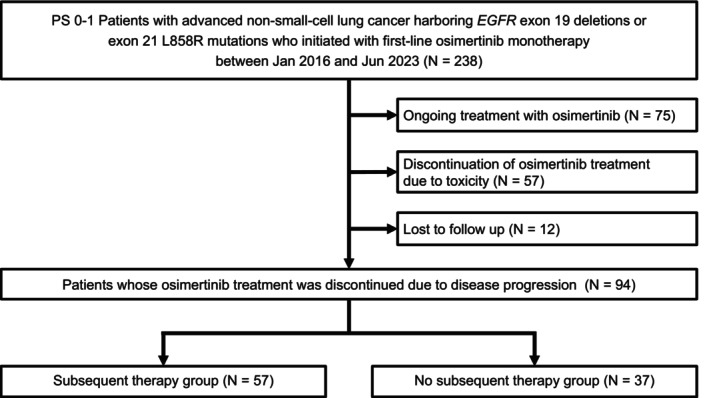
Flowchart of all patients. NST, non‐subsequent therapy; ST, subsequent therapy.

A total of 238 patients received first‐line osimertinib during the study period. We excluded 75 patients who were still undergoing osimertinib treatment at the data cutoff, 57 who discontinued osimertinib due to toxicity, and 12 who were lost to follow‐up. Ultimately, 94 patients who experienced disease progression during osimertinib monotherapy were included in the study. Of these, 57 patients (60.6%) received subsequent therapy (ST group), whereas 37 (39.4%) did not receive further treatment (NST group) (Figure 1).

Baseline characteristics of the patients at the time of initiation of osimertinib monotherapy are shown in Table 1. The ST and NST groups were similar across variables, including sex, PS at the time of initiation of osimertinib monotherapy, *EGFR* mutation subtype, and smoking history. However, the median age was 69 years (range, 31–81) in the ST group and 75 years (range, 49–88) in the NST group, with a significantly higher proportion of patients aged > 75 years (24.6% vs. 51.4%, *p* = 0.01). Notably, baseline central nervous system (CNS) metastases were present in 14 patients (24.6%) in the ST group and 20 patients (54.1%) in the NST group, occurring significantly more often in the NST group (*p* < 0.01). Among those with baseline CNS metastasis, five in the ST group and seven in the NST group received radiation therapy, while one patient in each group underwent surgery before receiving osimertinib monotherapy.

**TABLE 1:** Background characteristics at initiation of osimertinib.

	ST group (*N* = 57), *N* (%)	NST group (*N* = 37), *N* (%)	*p*
Age, median [range]	69 [31–81]	75 [49–88]	< 0.01
Age			0.01
≥ 75	14 (24.6)	19 (51.4)	
< 75	43 (75.4)	18 (48.6)	
Sex			1
Male	19 (33.3)	12 (32.4)	
Female	38 (66.7)	25 (67.6)	
PS			0.63
0	15 (26.3)	8 (21.6)	
1	42 (73.7)	29 (78.4)	
*EGFR* mutation			0.21
Exon 19 deletion	31 (54.4)	15 (40.5)	
L858R mutation	26 (45.6)	22 (59.5)	
History of smoking			1
Yes	26 (45.6)	17 (45.9)	
No	31 (54.4)	20 (54.1)	
Stage			1
IV	42 (73.7)	27 (73.0)	
Relapse	15 (26.3)	10 (27.0)	
Pleural effusion			0.68
Yes	26 (45.6)	15 (40.5)	
No	31 (53.4)	22 (59.5)	
CNS metastasis			< 0.01
Yes	14 (24.6)	20 (54.1)	
No	43 (75.4)	17 (45.9)	
Liver metastasis			1
Yes	10 (17.5)	6 (16.2)	
No	47 (82.5)	31 (83.8)	
Bone metastasis			0.83
Yes	23 (40.4)	14 (37.8)	
No	34 (59.6)	23 (62.2)	

Abbreviations: CNS, central nervous system; *EGFR*, epidermal growth factor receptor, NST, non‐subsequent therapy; PS, performance status; ST, subsequent therapy.

Univariate and multivariate analysis identified age > 75 years (odds ratio [OR], 2.98; 95% confidence interval [CI]: 1.19–7.48, *p* = 0.02) and baseline CNS metastasis (OR, 3.35; 95% CI: 1.35–8.35, *p* < 0.01) as independent risk factors for not receiving subsequent therapy (Table 2).

**TABLE 2:** Clinical risk factors for non‐subsequent therapy.

	*N*	Odds ratio (95% CI)	*p*
*Univariate analysis*			
Age	(≧ 75/75 <) 33/61	3.24 (1.34–7.84)	< 0.01
Sex	(Male/female) 31/63	1.04 (0.43–2.52)	0.93
PS	(1/0) 71/23	0.77 (0.29–2.06)	0.61
*EGFR* mutation	(L858R/19 del) 48/56	0.57 (0.24–1.32)	0.19
History of smoking	(Yes/no) 43/51	0.98 (0.43–2.26)	0.98
Stage	(IV/relapse) 69/25	0.96 (0.38–2.46)	0.94
Pleural effusion	(Yes/no) 41/53	0.81 (0.35–1.88)	0.63
CNS metastasis	(Yes/no) 34/60	3.61 (1.49–8.75)	< 0.01
Liver metastasis	(Yes/no) 16/78	0.91 (0.30–2.76)	0.87
Bone metastasis	(Yes/No) 37/57	0.90 (0.38–2.10)	0.81
*Multivariate analysis*			
Age ≧ 75		2.98 (1.19–7.48)	0.02
CNS metastasis		3.35 (1.35–8.35)	< 0.01

Abbreviations: CI, confidence interval; CNS, central nervous system; *EGFR*, epidermal growth factor receptor; PS, performance status.

### 3.2 Treatment Efficacy of Osimertinib

**FIGURE 2:**
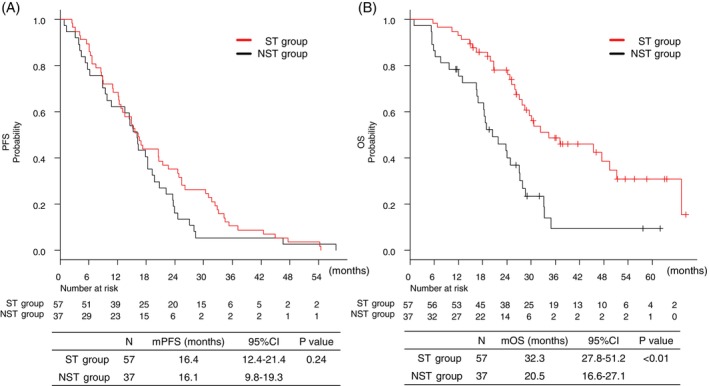
Kaplan–Meier curves for (A) progression‐free survival and (B) overall survival in the osimertinib monotherapy group. CI, confidence interval; mOS, median overall survival; mPFS, median progression‐free survival; NST, non‐subsequent therapy; ST, subsequent therapy.

With a median follow‐up of 41.5 months, the median duration of osimertinib treatment was 16.5 months (range, 2.0–54.4) in the ST group and 17.8 months (range, 0.82–48.0) in the NST group. The efficacy of first‐line osimertinib did not differ between the groups. The overall response rate was 63.2% in the ST group and 56.8% in the NST group (*p* = 0.71). The median PFS was 16.4 months (95% CI: 12.4–21.4) in the ST group and 16.1 months (95% CI: 9.8–19.3) in the NST group (*p* = 0.24) (Figure 2A). In contrast, OS was significantly longer in the ST group: 32.3 months (95% CI: 27.8–51.2) compared with 20.5 months (95% CI: 16.6–27.1) in the NST group (*p* < 0.01) (Figure 2B).

### 3.3 Characteristics at Osimertinib Progression and Subsequent Treatment

At the time of disease progression during osimertinib monotherapy, the median age was 70 years (range, 33–82 years) in the ST group and 76 years (range, 50–90 years) in the NST group (*p* < 0.01). While 93% of patients in the ST group maintained a PS of 0–1 at progression, more than 80% of patients in the NST group experienced a decline in PS. Specifically, the proportion of patients with a PS score ≥ 3 at progression was 0% in the ST group and 64.9% in the NST group (*p* < 0.01).

Progression patterns during osimertinib monotherapy are summarized in Table 3. A significantly higher proportion of patients in the ST group experienced intrathoracic disease progression, compared with those in the NST group (94.7% vs. 70.3%, *p* < 0.01). In contrast, CNS metastasis progression was more common in the NST group (29.7% vs. 14.0%, *p* = 0.07). The incidence of newly emergent meningitis was significantly higher in the NST group (21.6% vs. 1.8%, *p* < 0.01), compared with the ST group. Among the 11 patients in the NST group who had CNS metastasis progression, 4 (36.4%) experienced worsening of baseline CNS lesions, while 7 (63.6%) developed new CNS metastases; all 7 patients subsequently developed meningitis.

**TABLE 3:** Sites of disease progression during osimertinib monotherapy.

	ST group (*N* = 57), *N* (%)	NST group (*N* = 37), *N* (%)	*p*
Intrathoracic			< 0.01
Yes	54 (94.7)	26 (70.3)	
No	3 (5.3)	11 (29.7)	
CNS metastasis			0.07
Yes	8 (14.0)	11 (29.7)	
No	49 (86.0)	26 (70.3)	
Meningitis			< 0.01
Yes	1 (1.8)	8 (21.6)	
No	56 (98.2)	29 (78.4)	
Liver metastasis			0.43
Yes	3 (5.3)	4 (10.8)	
No	54 (94.7)	33 (89.2)	
Bone metastasis			0.35
Yes	9 (15.8)	3 (8.1)	
No	48 (84.2)	34 (91.9)	

Abbreviations: CNS, central nervous system; NST, non‐subsequent therapy; ST, subsequent therapy.

After disease progression during osimertinib monotherapy was confirmed, 27 of the 94 patients (28.7%) continued osimertinib monotherapy beyond progression. This included 21 of 80 patients (26.3%) with intrathoracic progression, 11 of 19 (57.9%) with CNS metastasis progression, 6 of 9 (66.7%) with meningitis progression, 4 of 7 (57.1%) with liver metastasis progression, and 4 of 12 (33.3%) with bone metastasis progression. There was no significant difference in the rate of osimertinib continuation between the ST and NST groups (22.8% vs. 37.8%, *p* = 0.16).

Of the 57 patients in the ST group, 10 were enrolled in clinical trials. The remaining 47 received post‐progression treatment in clinical practice: 38 received platinum‐based combination chemotherapy, 7 received platinum‐based chemotherapy plus immune checkpoint inhibitors, and 2 received other cytotoxic therapies.

### 3.4 Reasons for Discontinuation of Subsequent Therapy in the NST Group

In the NST group, the primary reason for not receiving second‐line therapy after osimertinib progression was deterioration in PS (89%), followed by patient refusal (8%) and age‐related dementia (3%) (Table 4). Among those with PS decline, the leading causes were respiratory deterioration characterized by desaturation and dyspnea (46%), CNS metastasis progression (27%), and cachexia‐like symptoms such as fatigue and anorexia (24%) (Table 4). Respiratory decline was mainly due to pleural effusion, and pulmonary embolism was caused by tumor‐related coagulation abnormality.

**TABLE 4:** Reasons for selecting non‐subsequent therapy.

	All NST cases (*N* = 37)	Age ≧ 75 (*N* = 19), *N* (%)	Age < 75 (*N* = 18), *N* (%)	With CNS metastasis (*N* = 20), *N* (%)	Without CNS metastasis (*N* = 17), *N* (%)
Deterioration of PS	33 (89.0%)	18 (94.7%)	15 (88.9%)	19 (95.0%)	14 (82.4%)
Respiratory decline	15 (45.5%)	10 (52.6%)	5 (27.8%)	6 (30.0%)	9 (64.3%)
CNS progression	9 (27.3%)	2 (10.5%)	7 (38.9%)	8 (40.0%)	1 (7.1%)
Cachexia‐like symptom	8 (24.2%)	5 (26.3%)	3 (16.7%)	5 (25.0%)	3 (21.4%)
Bone metastasis	1 (3.0%)	1 (5.3%)	0 (0.0%)	0 (0.0%)	1 (7.1%)
Others^a^	4 (12.1%)	1 (5.3%)	3 (16.7%)	1 (5.0%)	3 (11.8%)
Patients' refusal	3 (8.1%)	0 (0.0%)	3 (16.7%)	1 (5.0%)	2 (11.1%)
Age‐dependent dementia	1 (3.0%)	1 (5.3%)	0 (0.0%)	0 (0.0%)	1 (5.9%)

Abbreviations: CNS, central nervous system; NST, non‐subsequent therapy; PS, performance status.

^a^ Include patients' refusal and age‐dependent dementia.

Among patients aged ≥ 75 years and those aged < 75 years in the NST group, the predominant reason for discontinuing second‐line therapy was deterioration in PS. However, the underlying causes of PS decline varied by age group. Respiratory deterioration was more common in patients aged ≥ 75 years, affecting 10 of 19 (52.6%), compared with 5 of 18 (27.8%) among those aged < 75 years. Conversely, CNS metastasis progression was more prevalent in the younger subgroup, occurring in 7 of 18 patients (38.9%) compared with 2 of 19 (10.5%) in the older subgroup.

Regardless of baseline CNS metastasis status, PS deterioration remained the primary reason for ineligibility for second‐line therapy. Among the 20 patients with baseline CNS metastases, CNS progression was the leading cause of PS decline, occurring in 8 (40.0%). In contrast, among the 17 patients without baseline CNS metastases, only 1 (7.1%) experienced CNS progression contributing to PS decline.

## 4 Discussion

In this study, we aimed to identify patient subgroups that may be disadvantaged by first‐line osimertinib monotherapy. We found that nearly 40% of patients were unable to proceed to second‐line treatment, a rate consistent with previous reports [1, 3]. These patients were significantly older, had a higher incidence of baseline CNS metastasis, and were more likely to experience CNS metastasis progression—particularly the development or worsening of meningitis. Interestingly, the PFS and objective response rate of osimertinib monotherapy were compared between the ST and NST groups, indicating that the discontinuation of subsequent therapy was likely not due to the efficacy of osimertinib itself but rather to baseline patient characteristics, which may have contributed to PS deterioration at progression. Nearly 60% of older patients aged > 75 years (19/33, 57.6%) and nearly 60% of those with baseline CNS metastases (20/34, 58.8%) were unable to transit to subsequent therapy. Multivariate analysis confirmed advanced age and baseline CNS metastases as independent risk factors for failing to receive further treatment. For such patients, osimertinib monotherapy may be suboptimal, and alternative treatment strategies are warranted.

Brain metastases occur in approximately 20%–40% of patients with NSCLC, with even higher rates observed among those with *EGFR* mutations [7, 8, 9, 10]. Accordingly, effective management of CNS metastases is critical in the treatment of *EGFR*‐mutated NSCLC. Compared with first‐generation EGFR‐TKIs, osimertinib demonstrates superior blood–brain barrier penetration and has been reported to achieve intracranial tumor control rates of 75%–100% [11, 12, 13, 14]. Nevertheless, the NST group had a significantly higher rate of baseline CNS metastases, and 58.8% of these patients could not proceed to subsequent treatment—a proportion notably higher than that of the overall study population (39.4%). Furthermore, among patients with baseline CNS metastases, 40% experienced CNS metastasis progression (particularly meningitis), which led to PS decline and precluded subsequent therapy. In contrast, the incidence of new CNS metastases in patients without baseline CNS involvement was only 5%. Previous studies have reported similar trends, suggesting that while osimertinib effectively prevents new CNS metastases, its ability to control pre‐existing CNS disease may be insufficient [13].

The observation that nearly 60% of patients with baseline CNS metastases could not transition to subsequent treatment underscores the need for improved therapeutic strategies in this subgroup. Several approaches have been considered for managing CNS metastases in advanced *EGFR*‐mutated NSCLC. A recently presented pooled analysis and meta‐analysis found insufficient evidence to suggest that upfront radiation therapy improves survival in *EGFR*‐mutant patients with brain metastases [15, 16]. However, several studies have demonstrated that upfront radiation therapy may be an effective option, especially for patients with a small number of brain metastases, indicating its potential and optimal use [17, 18]. Also, the effectiveness of osimertinib in combination with angiogenesis inhibitors has been reported to be controversial; however, it might also offer potential benefits in cases with CNS metastasis [19, 20, 21, 22]. On the other hand, in the FLAURA2 study, adding chemotherapy to osimertinib significantly prolonged CNS PFS in patients with existing CNS metastasis by 24.9 months compared with 13.8 months with osimertinib monotherapy [3]. Similarly, the MARIPOSA study demonstrated that twice as many patients remained free of intracranial progression at 3 years, with a longer intracranial PFS of 25.4 months compared with 22.2 months with osimertinib [23]. While an optimal treatment selection algorithm has not yet been established, therapies that combine radiation or chemotherapy may offer alternative approaches for patients with baseline CNS metastases.

Advanced age is a well‐established risk factor for adverse events associated with anticancer therapy and further exacerbates treatment vulnerability [24, 25, 26]. In our study, cessation of subsequent therapy among older patients was primarily due to respiratory deterioration, presumably caused by age‐related comorbidities and cancer progression. Notably, patients who discontinued osimertinib treatment due to toxicity were excluded from this study; therefore, the true proportion of those ineligible for second‐line therapy may be underestimated. Consistent with our findings, real‐world data from Japan reported that only 39% of patients aged > 75 years transitioned to second‐line treatment following osimertinib progression [27]. This retrospective study also showed that older patients exhibited a higher rate of treatment discontinuation due to adverse events, resulting in a shorter time to treatment failure, despite PFS being comparable to that of patients aged < 75 years. Some older patients were unable to proceed to subsequent treatment because, in addition to baseline physical vulnerability, the toxicity of osimertinib—although not severe—was sufficient to worsen their PS when combined with disease progression. Therefore, for older patients, developing less toxic therapies may be more beneficial than pursuing newer agents that offer greater efficacy but also carry increased toxicity.

Our study was limited by its small sample size and the recruitment of patients from a single center. Furthermore, because this was a retrospective study, the rationale for selecting best supportive care was based on the treating physician's documentation, which may not have fully captured the underlying reason, particularly when multiple factors contributed to treatment cessation. The frequency of computed tomography and magnetic resonance imaging was determined at each physician's discretion, introducing the possibility that more intensive surveillance and earlier intervention could have enabled more patients to proceed to subsequent treatment.

## 5 Conclusion

In patients with advanced *EGFR*‐mutated NSCLC, baseline CNS metastasis and advanced age are significant barriers to receiving second‐line therapy. Refinements in first‐line treatment strategies are needed—either to improve transition rates to ST or to maximize the duration of benefit from initial treatment when continuation is not feasible. Prospective studies are warranted to validate these findings and to explore alternative first‐line or combination strategies for patients with CNS metastases or advanced age.

## Author Contributions

**Hiroaki Kodama:** writing – original draft. **Haruyasu Murakami:** conceptualization, project administration, investigation, writing – review and editing. **Nobuaki Mamesaya:** resources, writing – review and editing. **Haruki Kobayashi:** resources, writing – review and editing. **Kazushige Wakuda:** resources, writing – review and editing. **Ryo Ko:** resources, writing – review and editing. **Akira Ono:** resources, writing – review and editing. **Tateaki Naito:** resources, writing – review and editing. **Hirotsugu Kenmotsu:** resources, review and editing. **Tetsuo Shimizu:** writing – review and editing. **Yasuhiro Gon:** writing – review and editing; **Toshiaki Takahashi:** resources, writing – review and editing.

## Ethics Statement

This study was approved by the Institutional Review Board of Shizuoka Cancer Center (J2024‐224‐2024‐1‐3) and was conducted in accordance with the principles of the Declaration of Helsinki.

## Consent

Informed consent was obtained using an opt‐out approach via the hospital website and posted notices. Patients who declined participation were excluded.

## Conflicts of Interest

Hiroaki Kodama received personal fees from Chugai Pharma, Daiichi Sankyo, and Novartis Pharma K.K. outside of the submitted work. H.M. received grants and personal fees from AstraZeneca K.K., Takeda Pharmaceutical Co. Ltd., Daiichi‐Sankyo Co., Chugai Pharmaceutical Co. Ltd., and Taiho Pharmaceutical; grants from AbbVie, IQvia, and Bayer; and personal fees from Ono Pharmaceutical Co. Ltd., Amgen, Eli Lilly Japan K.K., Novartis Pharma K.K., Pfizer Inc., Bristol‐Myers Squibb Japan, MSD K.K., Eisai, and Nihon Kayaku, outside of the submitted work. N.M. received grants and personal fees from Boehringer Ingelheim and personal fees from Taiho Pharmaceuticals, Chugai Pharmaceutical Co. Ltd., AstraZeneca K.K., MSD K.K., and Ono Pharmaceutical Co. Ltd. outside of the submitted work. Haruki Kobayashi received personal fees from Novartis Pharma K.K., Ono Pharmaceutical Co. Ltd., Taiho Pharmaceutical Co., AstraZeneca K.K., Eli Lilly K.K., Bristol‐Myers Squibb Company, Chugai Pharmaceutical Co. Ltd., and Daiichi Sankyo Co. outside of the submitted work. R.K. received grants and personal fees from AstraZeneca K.K. and MSD K.K. and personal fees from Ono Pharmaceutical, Daiichi Sankyo, Chugai Pharmaceutical Co. Ltd., Eli Lilly K.K., Taiho Pharmaceuticals, and Takeda outside of the submitted work. K.W. received grants and personal fees from MSD K.K., AstraZeneca K.K., Chugai Pharmaceutical Co. Ltd., and Daiichi Sankyo Co. Ltd.; grants from AbbVie, Novartis Pharma K.K.; AMGEN, and Dizal Pharma; and personal fees from Taiho Pharmaceutical, Boehringer Ingelheim, Nihon Kayaku Janssen Pharmaceutical K.K., Ono Pharmaceutical, Takeda Pharmaceutical, and Eli Lilly K.K. outside of the submitted work. A.O. received personal fees from AstraZeneca K.K., Chugai Pharmaceutical Co. Ltd., Ono Pharmaceutical Co. Ltd., and Indica Laboratories outside of the submitted work. Hirotsugu Kenmotsu received personal fees from AMGEN, Bayer, Boehringer Ingelheim, Chugai Pharmaceutical Co., Bristol‐Myers Squibb, Daiichi‐Sankyo Co. Ltd., Kyowa Hakko Kirin Co. Ltd., Taiho Pharmaceuticals, Takeda Pharmaceutical Co. Ltd., Merck Biopharma Co. Ltd., MSD K.K., and Pfizer; grants and personal fees from AstraZeneca K.K., Novartis Pharma K.K., Ono Pharmaceutical Co. Ltd., and Eli Lilly K.K.; and grants from Loxo Oncology outside of the submitted work. T.N. received grants from Otsuka Pharmaceutical K.K. and Japan's Agency for Medical Research and Development (AMED) and personal fees from Ono Pharmaceutical Co. Ltd., and Helsinn Healthcare SA outside of the submitted work. T.S. received grants and personal fees from Ono Pharmaceutical Co. Ltd. and Chugai Pharmaceutical Co. Ltd.; grants from Eli Lilly K.K.; and personal fees from AstraZeneca K.K., MSD, and Taiho Pharmaceuticals outside of the submitted work. T.T. received grants and personal fees from MSD, Chugai Pharmaceutical Co. Ltd., Pfizer Japan Inc., Eli Lilly Japan K.K., AstraZeneca K.K., and Amgen Inc.; personal fees from Takeda Pharmaceuticals Co., Ono Pharmaceutical Co. Ltd., BMS Japan, and Novartis; and grants from Janssen Pharmaceutical K.K., Merck Biopharma Co. Ltd., and An Heart Therapeutics Inc. outside of the submitted work. The other authors declare no conflicts of interest.

## Data Availability

The data that support the findings of this study are available from the corresponding author upon reasonable request.
